# First report of acute African swine fever in pig farms on the west coast of India: pathological and molecular insights

**DOI:** 10.3389/fcimb.2025.1710337

**Published:** 2025-10-28

**Authors:** Shirish Dadarao Narnaware, Susitha Rajkumar, Prasastha Vemula, Sanjay V. Udharwar, Amiya Ranjan Sahu

**Affiliations:** ^1^ Animal and Fisheries Science Section, ICAR- Central Coastal Agricultural Research Institute, Old Goa, Goa, India; ^2^ Krishi Vigyan Kendra, ICAR- Central Coastal Agricultural Research Institute, Old Goa, Goa, India

**Keywords:** African swine fever, India, pathology, phylogenetic analysis, p72, P54

## Abstract

**Background:**

African swine fever (ASF) represents one of the most devastating viral threats to global pig production, with mortality often approaching 100% and severe socioeconomic consequences. India confirmed its first ASF outbreaks in 2020 in the north-eastern states, but no cases had previously been documented on the west coast. This study presents the first confirmed ASF outbreaks in pig farms on India’s west coast, providing clinicopathological and molecular insights.

**Materials:**

Between January and June 2025, two pig farms in North Goa experienced unusually high mortalities. The affected pigs were clinically examined, and necropsies were performed on eight carcasses for detailed pathological analysis. Tissue samples (spleen, kidneys, lungs, liver, and lymph nodes) and blood were collected for histopathology and polymerase chain reaction (PCR)-based diagnosis. African swine fever virus (ASFV) was detected using PCR targeting the *B646L* gene, which encodes the major capsid protein p72, for molecular confirmation. For genotyping, the partial *B646L* (p72) gene and the complete *E183L* (p54) gene were amplified, sequenced, and subjected to phylogenetic analysis.

**Results:**

Both farms exhibited extremely high mortality (95%–96%), with affected pigs showing fever, depression, cyanosis, respiratory distress, and widespread cutaneous hemorrhages. Gross pathology revealed splenomegaly, hepatomegaly, severely congested kidneys with petechial hemorrhages, hemorrhagic lymphadenopathy, and vascular congestion. Histopathology demonstrated severe lymphoid depletion, necrosis, and hemorrhages in the spleen and lymph nodes, along with glomerulonephritis in the kidneys. ASFV was confirmed by PCR in all samples. Phylogenetic analysis placed the isolates in genotype II, showing complete identity with earlier Indian and Asian strains, indicating continued circulation of this lineage in the region.

**Conclusion:**

The emergence of ASF in Goa highlights the spread of genotype II to India’s west coast, underscoring the urgent need to strengthen farm biosecurity, raise farmer awareness, and implement active surveillance to protect the expanding pig sector from severe economic losses.

## 1 Introduction

African swine fever (ASF) is a highly contagious, notifiable, transboundary disease of domestic pigs (*Sus scrofa domesticus*) and wild suids, leading to severe mortality. ASF is caused by a large, enveloped, double-stranded DNA virus of the genus *Asfivirus* within the family *Asfarviridae*. The virus is unique among DNA viruses because it replicates in the cytoplasm of infected cells, primarily targeting monocytes and macrophages (Dixon et al., 2013). The World Organization for Animal Health (WOAH) classifies ASF as a notifiable transboundary animal disease due to its rapid spread, high mortality rates, and significant socioeconomic consequences (WOAH, 2024a). ASF has emerged as a major global threat to pig production, food security, and livelihoods in all the pig-rearing countries. Since its initial discovery in Kenya in the 1920s, ASF remained endemic in parts of sub-Saharan Africa until its introduction into Europe in the late 1950s, and later into the Caucasus and Asia, causing devastating losses in all countries with significant pig farming industries (Penrith, 2009; Khomenko et al., 2025; Coelho et al., 2025). The 2018 incursion of ASF into China—the world’s largest pig producer—marked a turning point in its global spread, with subsequent dissemination into Southeast Asia and India (Zhou et al., 2018). India reported its first outbreaks of ASF in Assam and Arunachal Pradesh during February–March 2020, predominantly affecting backyard pig populations and marking a notable expansion in the disease’s epidemiological landscape (Dutta et al., 2021; Rajukumar et al., 2021). Since then, sporadic occurrences have been documented across the north-eastern states and, more recently, in the southern and northern regions of the country (Rajkhowa et al., 2022; Hiremath et al., 2024; Verma et al., 2024). Until now, however, no cases had been reported from the western coastal region, where pig farming is comparatively less intensive but has been steadily increasing in peri-urban and rural areas.

Transmission of the disease primarily occurs through close contact, ingestion of contaminated feed, exposure to infected fomites, and the involvement of soft ticks of the genus *Ornithodoros* in both domestic pigs and wild boars (Galindo and Alonso, 2017; Dixon et al., 2020). Major risk factors for disease spread include live pig movement, swill feeding, contaminated feed and vehicles, and human-mediated transmission (Cheng and Ward, 2022). However, the specific risk factors driving ASF transmission in India remain poorly defined (Hiremath et al., 2024). The country’s diverse geography and heterogeneous pig husbandry practices across states highlight the need for detailed outbreak investigations to identify circulating ASFV subgroups and their associated risk determinants.

Clinically, ASF manifests in peracute, acute, subacute, and chronic forms, depending on the virulence of the virus and host factors (Li et al., 2022). The acute form is most commonly observed during outbreaks involving highly virulent strains and is characterized by the sudden onset of high fever (40.5°C–42°C), anorexia, depression, skin hyperemia or cyanosis, respiratory distress, and high mortality (approaching 100%) within a short incubation period (Sánchez-Vizcaíno et al., 2015; Salguero, 2020). In domestic swine, the pathogenesis of African swine fever is primarily marked by severe lymphoid depletion leading to lymphopenia and immunosuppression, accompanied by widespread hemorrhages across multiple organs (Salguero, 2020). African wild suids, such as bushpigs (*Potamochoerus larvatus*), red river hogs (*Potamochoerus porcus*), and warthogs (*Phacochoerus africanus*), can become infected with ASFV without exhibiting clinical disease, thereby serving as natural reservoir hosts (Salguero, 2020). Post-mortem findings typically include splenomegaly, hemorrhagic lymphadenitis, petechial hemorrhages in multiple organs, and serous or hemorrhagic effusions (Galindo and Alonso, 2017). In endemic regions, less virulent strains may cause chronic disease, characterized by variable general clinical signs, including skin necrosis, arthritis, growth retardation, emaciation, respiratory distress, and abortion, which complicates diagnosis and control (Salguero, 2020; Li et al., 2022).

Although ASF is a highly contagious and deadly viral disease, no vaccine or treatment is currently available for its prevention. Efforts to develop an effective ASF vaccine are hindered by an incomplete understanding of ASFV biology, viral pathogenesis, and immune evasion mechanisms, as well as the complexity of its large genome (Chandana et al., 2024). Gene-deleted live attenuated vaccines approved in Vietnam in 2023—AVAC ASF LIVE (ASFV-G-ΔMGF) and NAVET-ASFVAC (ASFV-G-ΔI177L)—were later discontinued due to biosafety concerns, while viral vector vaccines have failed to provide sufficient protection (Diep et al., 2024; Yang et al., 2025). Nevertheless, recent trials in Vietnam evaluating the safety and efficacy profiles of AVAC ASF LIVE demonstrated high immunogenicity and strong protection against genotype II ASFV infection (Diep et al., 2025). In the Philippines, trials of the AVAC ASF LIVE vaccine demonstrated that all vaccinated pigs (4–10 weeks old) developed antibodies against ASFV and remained free from clinical disease and viral shedding (Fernandez-Colorado et al., 2024). Based on these findings, the Philippine government is currently procuring ASF vaccines to initiate widespread vaccination. While ASF control remains a long-term global challenge, the successful commercialization and field application of ASF vaccines in Vietnam and the Philippines provide optimism for disease containment. Continued development and approval of vaccines effective against virulent field strains are expected to advance efforts to mitigate the economic impact of ASF worldwide. The disease causes significant economic losses due to high mortality rates (up to 100%), culling policies, trade restrictions, and disruption of pig supply chains (WOAH, 2019; Salguero, 2020). It is estimated that the 2018–2020 ASF pandemic led to the loss of over 50% of China’s pig population, resulting in a global spike in pork prices and food inflation (Ma et al., 2021). In India, where pig farming supports the livelihoods of millions, especially in tribal and rural communities, ASF outbreaks have already caused losses exceeding INR 100 crore in north-eastern states (Mohan et al., 2021). The detection of ASF in a previously uninfected region, such as the west coast, raises urgent concerns regarding disease containment and the protection of pig farmers.

Accurate and rapid diagnosis of ASF is critical for disease control. Confirmatory diagnosis is typically achieved through polymerase chain reaction (PCR), which targets conserved genes such as *B646L* encoding p72, a structural protein essential for virus classification (King et al., 2003). Real-time PCR and conventional PCR methods recommended by WOAH are widely used for field and laboratory confirmation (WOAH, 2024b). Molecular characterization, including sequencing of the p72 and p54 genes and phylogenetic analysis, enables genotype assignment and comparison with known ASFV lineages (Achenbach et al., 2017; O’Dwyer et al., 2025). To date, 24 ASFV genotypes have been identified based on p72 gene analysis, with Genotype II responsible for recent outbreaks across Eurasia, including India (Gallardo et al., 2014; Rajukumar et al., 2021; Rajkhowa et al., 2022; Hiremath et al., 2024; O’Dwyer et al., 2025).

Pig farming on the west coast of India is a growing but relatively underdeveloped sector compared to other regions of the country. In the west-coast region, pig production is predominantly small-scale and is often constrained by inadequate biosecurity, the practice of swill feeding, and unregulated trade networks, collectively creating conditions conducive to ASF introduction and dissemination. Along the west coast, Goa is the smallest state in India, having a total pig population of around 35,480 pigs, which primarily includes the native Agonda breed, the exotic Large White Yorkshire (LWY) breed, and a large share of crossbred pigs (20th Livestock Census, 2019). This study reports the first confirmed outbreak of acute African swine fever on two pig farms located on the west coast of India, marking a significant epidemiological event with implications for national surveillance and control strategies. The objectives were to document the clinical presentation, gross and histopathological lesions, and to confirm the viral genotype through sequencing and phylogenetic analysis, thereby identifying the genetic lineage and potential origin of the outbreak strain in a region previously considered low-risk.

## 2 Materials and methods

### 2.1 Study area and outbreak history

**Figure 1:**
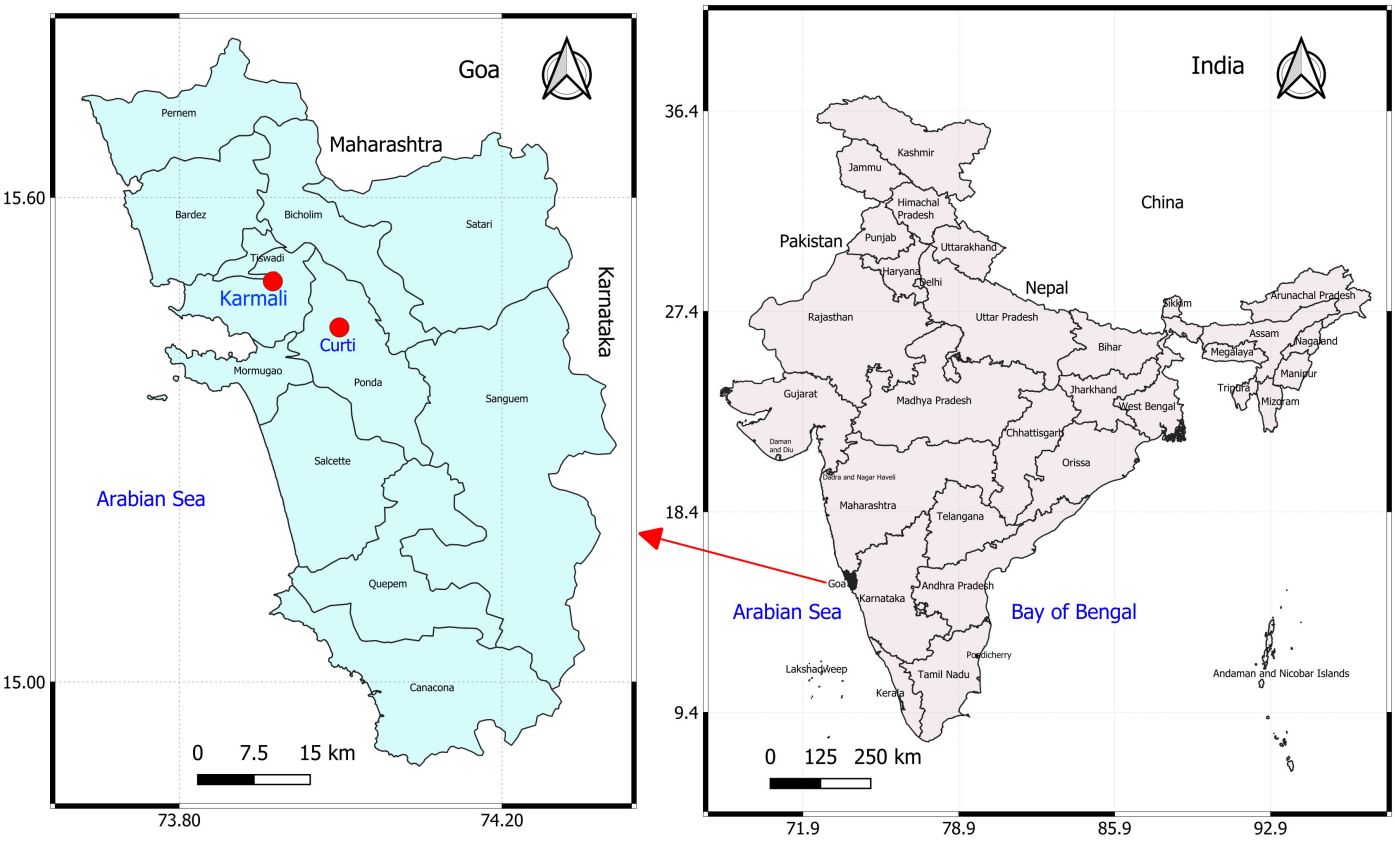
Map of Goa, a state on the west coast of India, showing the locations of affected pig farms (red dots).

Goa, located on the west coast of India, is the country’s smallest state, with a pig population of approximately 35,480, largely maintained through backyard and small-scale farming. The state is bordered by Maharashtra to the north, Karnataka to the south and east, and the Arabian Sea to the west. The region experiences a tropical monsoon climate, characterized by high humidity and heavy rainfall from June to September, followed by mild winters and hot summers. Temperatures generally range from 26°C to 36°C, with humidity between 60% and 80% (data from ICAR–Central Coastal Agricultural Research Institute, Old Goa Observatory). In the first week of January 2025, the first suspected ASF outbreak was reported on farm A in Karmali village, North Goa district (Latitude 15.480846, Longitude 73.923970) (**Figure 1**). The farm housed 234 pigs, including 154 adults and 80 piglets, primarily of local Agonda and crossbred types. Daily mortalities of one to five pigs, involving both adults and piglets, were observed over a period of 34 days. Prior to this outbreak, no cases of ASF or unusual mortality had been reported in pig farms in Goa. Farm history indicated limited biosecurity, poor hygiene, and swill feeding, occasionally including raw chicken skin from poultry slaughterhouses. The farm primarily marketed live piglets to other farmers for breeding and adult pigs to meat vendors for slaughter. A team of scientists from ICAR-CCARI, Goa, visited farm A 2 weeks after the first mortalities were reported. At the time of inspection, 20 pigs exhibited one or more clinical signs of the disease, and four pigs had died, on which necropsies were performed.

Farm B, an organized breeding unit located in Curti village, approximately 23 km from farm A, reported the first incidence of sudden pig mortalities in the second week of June 2025, nearly 6 months after the ASF outbreak in farm A. The farm maintained a total of 54 purebred LWY pigs (20 adults and 34 piglets) and experienced continuous daily losses of one to three pigs over a period of 20 days. Although the investigating team did not visit this farm, clinical observations, post-mortem examinations, and sampling were conducted by a veterinary officer from the Goa State Veterinary Department, who subsequently submitted tissue samples from four dead pigs for pathological and diagnostic investigations. The systematic investigation was conducted following the *Field Manual for Animal Disease Outbreak Investigation and Management* (OIE, 2018). Interviews with the farm owner and workers of both farms were conducted to collect data on management practices, clinical signs, and the number of affected cases and deaths. The affected pigs were assessed for clinical signs, overall health status, and the pattern of hemorrhagic skin lesions. After confirmation of ASF in the farms, the Goa State Veterinary Department was promptly informed, and national guidelines were followed for disease reporting and containment. In both farms, vaccination against classical swine fever was routinely implemented, and there was no evidence of tick infestation.

### 2.2 Pathological studies and sample collection

Carcasses of eight pigs, aged between 6 months and 2 years (four from each farm), were subjected to detailed necropsy. Gross lesions in various internal organs were recorded, and representative tissue samples from the lung, liver, kidney, spleen, heart, and lymph nodes were collected in 10% neutral buffered formalin for histopathology. Formalin-fixed tissues were processed, embedded in paraffin, sectioned at 5 µm, and stained with hematoxylin and eosin (H&E). Slides were examined and photographed under a semiautomated laboratory microscope (Leica DM3000 LED) equipped with a camera attachment (Leica DFC450 C).

### 2.3 Molecular diagnosis, sequencing, and phylogenetic analysis

Heart blood samples collected in EDTA vials, along with tissue samples including spleen, kidney, lung, and liver from eight necropsied pigs, were collected in sterile vials and transported under cold chain conditions to the laboratory for DNA isolation and molecular diagnosis. DNA extraction was carried out using a commercial kit (Thermo Scientific, USA GeneJET Genomic DNA Purification Kit) following the manufacturer’s protocol. For initial diagnosis, a PCR assay targeting the *B646L* gene, using the ASF diagnosis primers PPA1 (5′-AGTTATGGGAAACCCGACCC-3′) and PPA2 (5′-CCCTGAATCGGAGCATCCT-3′), which generates a 257-bp amplicon within the p72 protein, was used to confirm the presence of ASFV DNA (Agüero et al., 2003). For ASFV genotyping, partial p72 (*B646L*) and p54 (*E183L*) genes were amplified and sequenced. For p72 genotyping, the 478-bp C-terminal end of the *B646L* gene, encoding the major capsid protein p72, was amplified using the primers p72-U (5′-GGCACAAGTTCGGACATGT-3′) and p72-D (5′-GTACTGTAACGCAGCACAG-3′), as previously described (Bastos et al., 2003). To differentiate p72 genotype viruses from different regions and time periods, the complete *E183L* gene encoding the p54 protein was amplified using the primers PPA722 (5′-CGAAGTGCATGTAATAAACGTC-3′) and PPA89 (5′-TGTAATTTCATTGCGCCACAAC-3′), flanking a 683-bp DNA fragment (Gallardo et al., 2009).

Representative positive DNA amplicons for the p72 and p54 genes were sequenced using the Sanger method based on chain-terminating dideoxynucleotides (Barcode Biosciences, India). The sequences obtained were deposited in NCBI GenBank, and multiple sequence alignment was performed with the published sequences in NCBI Basic Local Alignment Search Tool (BLAST) (https://blast.ncbi.nlm.nih.gov/Blast.cgi). The nucleotide sequences of the p72 and p54 genes were aligned with other sequences from the NCBI GenBank database using the Clustal W tool. Phylogenetic trees were constructed using both the Neighbor-Joining and maximum likelihood methods, with the latter providing improved resolution and a more robust evolutionary analysis, employing the Tamura–Nei model in MEGA12 (Kumar et al., 2024). All the DNA samples were also tested by PCR against porcine circovirus 2 (PCV2) (Ellis et al., 1999), *Escherichia coli* (Heininger et al., 1999), *Pasteurella multocida* (Townsend et al., 1998), *Streptococcus* sp (Prabhu et al., 2013), and classical swine fever virus (CSFV) (Pan et al., 2005) to rule out possible co-infections.

## 3 Results

### 3.1 Clinical signs and gross pathology

**Figure 2:**
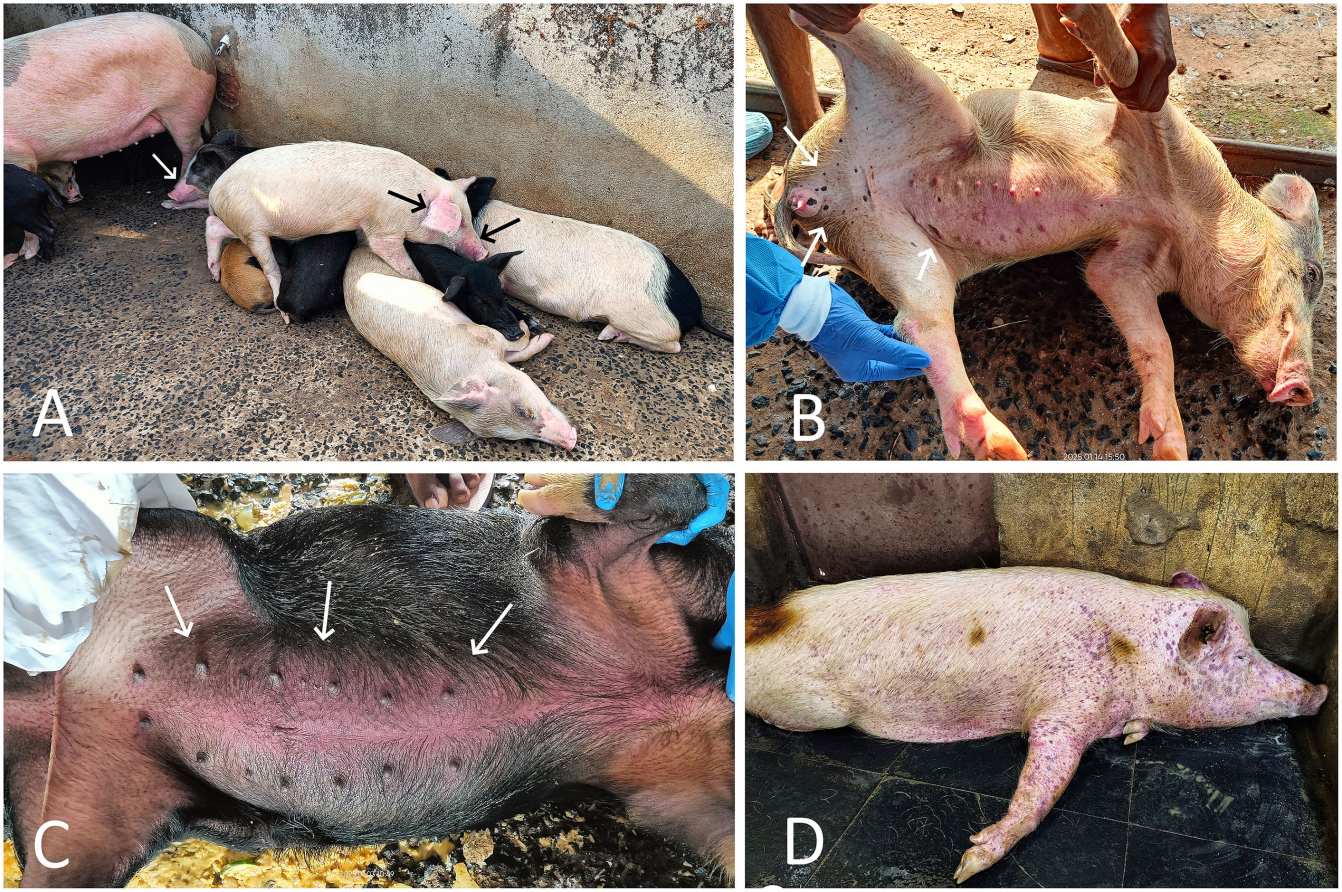
**(A)** ASF-affected local and crossbred pigs from farm A showing signs of huddling, lethargy, weakness, and cyanosis of the ears and snout (arrow). **(B)** ASF-affected crossbred pig from farm A showing scattered, variably sized black hemorrhagic spots (arrow) on the skin of the inguinal region and abdomen. **(C)** ASF-affected crossbred pig from farm A showing severe red discoloration of the abdominal skin (arrow). **(D)** ASF-affected Large White Yorkshire pig from farm B showing severe and widespread subcutaneous hemorrhages all over the body.

The overall mortality in farm A was recorded as 95.29% over a span of 34 days, with daily mortalities ranging from a minimum of one to a maximum of five pigs (including adults and piglets). The affected pigs exhibited one or more clinical signs of the disease, such as high fever (40°C–42°C), anorexia, depression, lethargy, huddling, cyanosis of the ears and snout, respiratory distress, and nasal discharge (**Figure 2A**). Among all the pigs, the white-colored crossbred pigs were found to be more severely affected and showed varying-sized, circumscribed, multiple black-colored spots of cutaneous ecchymoses, particularly in the inguinal area, inner thighs, legs, lower abdomen, and ventral surface of the body (**Figure 2B**). In black or dark-colored local and crossbred pigs, a reddish-colored diffuse area of congestion was observed, particularly in the ear, snout, and inguinal and abdominal regions, instead of hemorrhagic spots (**Figure 2C**). Abortion was recorded in two crossbred pregnant sows. On the day of the visit, a total of four pigs were found dead on the farm, on which necropsy was performed.

The overall mortality in farm B was recorded as 96.29%, with daily mortalities ranging from a minimum of one to a maximum of three pigs, including adults as well as piglets, over a span of 20 days. The clinical signs in affected pigs were largely similar to those in farm A; however, subcutaneous hemorrhages were more prominent and widespread throughout the entire body in the majority of affected LWY breed pigs on this farm (**Figure 2D**).

**Figure 3:**
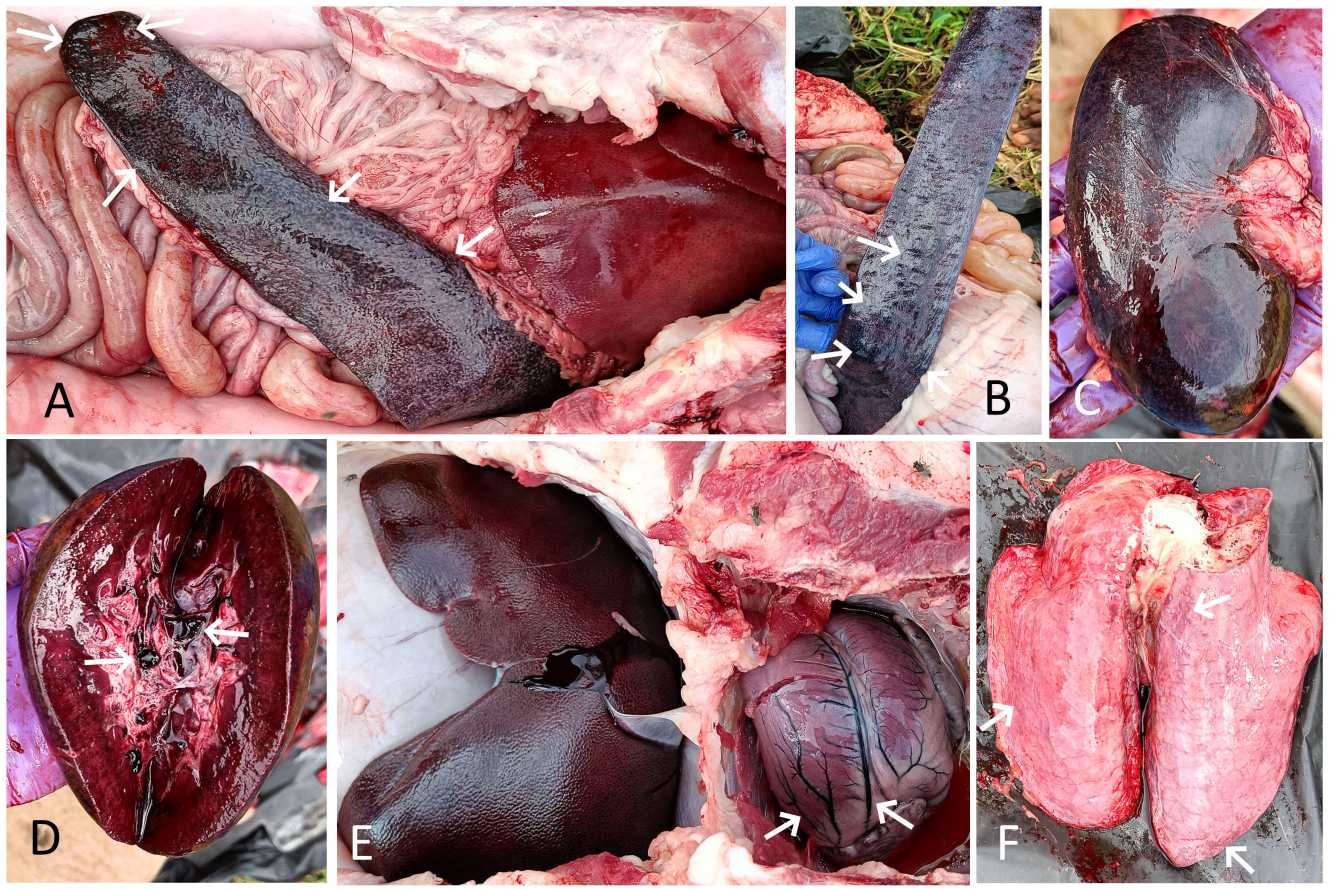
**(A)** Spleen of pig 1 showing splenomegaly, severe congestion, and dark-colored areas of infarction (arrow). The enlarged, dark red, diffusely congested liver is also visible. **(B)** Spleen of pig 2 showing multifocal, raised dark areas of infarction (arrow) and hemorrhages on the splenic surface (arrow). **(C)** Kidney of pig 1 showing severe diffuse congestion and hemorrhagic patches. **(D)** Cut section of the same kidney showing severe congestion and dark red blood clots in the renal pelvis (arrow). **(E)** Enlarged, dark, congested liver of pig 2. The enlarged heart with hyperemic blood vessels is also visible (arrow). **(F)** Lung of pig 2 showing edema, congestion, and petechial hemorrhages (arrow).

The necropsy of the affected dead pigs from both farms revealed consistent lesions of focal to diffuse hemorrhages and severe congestion in various internal organs, including the spleen, kidneys, lungs, liver, mesenteric lymph nodes, and intestines. Among all the internal organs, the most notable lesions were observed in the spleen and kidneys, which were severely affected in all eight necropsied pigs. The spleen was considerably enlarged, dark-colored, and severely congested, with round edges in all necropsied pigs. Multiple areas of infarction, appearing as raised, dark-colored patches of hemorrhages on the splenic surface, were also observed in four cases (**Figures 3A, B**). The kidneys of all infected dead pigs were dark red to dark brown, diffusely congested, contained dark red blood or blood clots in the renal pelvis, were soft, and had dark red petechial to ecchymotic hemorrhages on their surface (**Figures 3C, D**). The liver appeared enlarged in all the dead pigs and showed dark brown to black discoloration with diffuse congestion and hemorrhages at the edges (**Figures 3A, E**). The lungs of all the infected pigs failed to collapse, were enlarged, had mild to moderate edema, and showed multiple areas of consolidation in different lung lobes, along with petechial to ecchymotic hemorrhages (**Figure 3F**). Tracheal mucosa was moderately congested and contained a moderate amount of mucous mixed with froth in six cases. The heart showed mild to moderate enlargement with hyperemic blood vessels in all necropsied pigs (**Figure 3E**). Petechial hemorrhages on the epicardium were observed in three cases. The mucosa of the small intestine was moderately to severely congested and contained dark red hemorrhagic contents in five dead pigs. The mesenteric lymph nodes were enlarged and edematous in all pigs, with red patches of hemorrhages observed in three cases.

### 3.2 Histopathological findings

**Figure 4:**
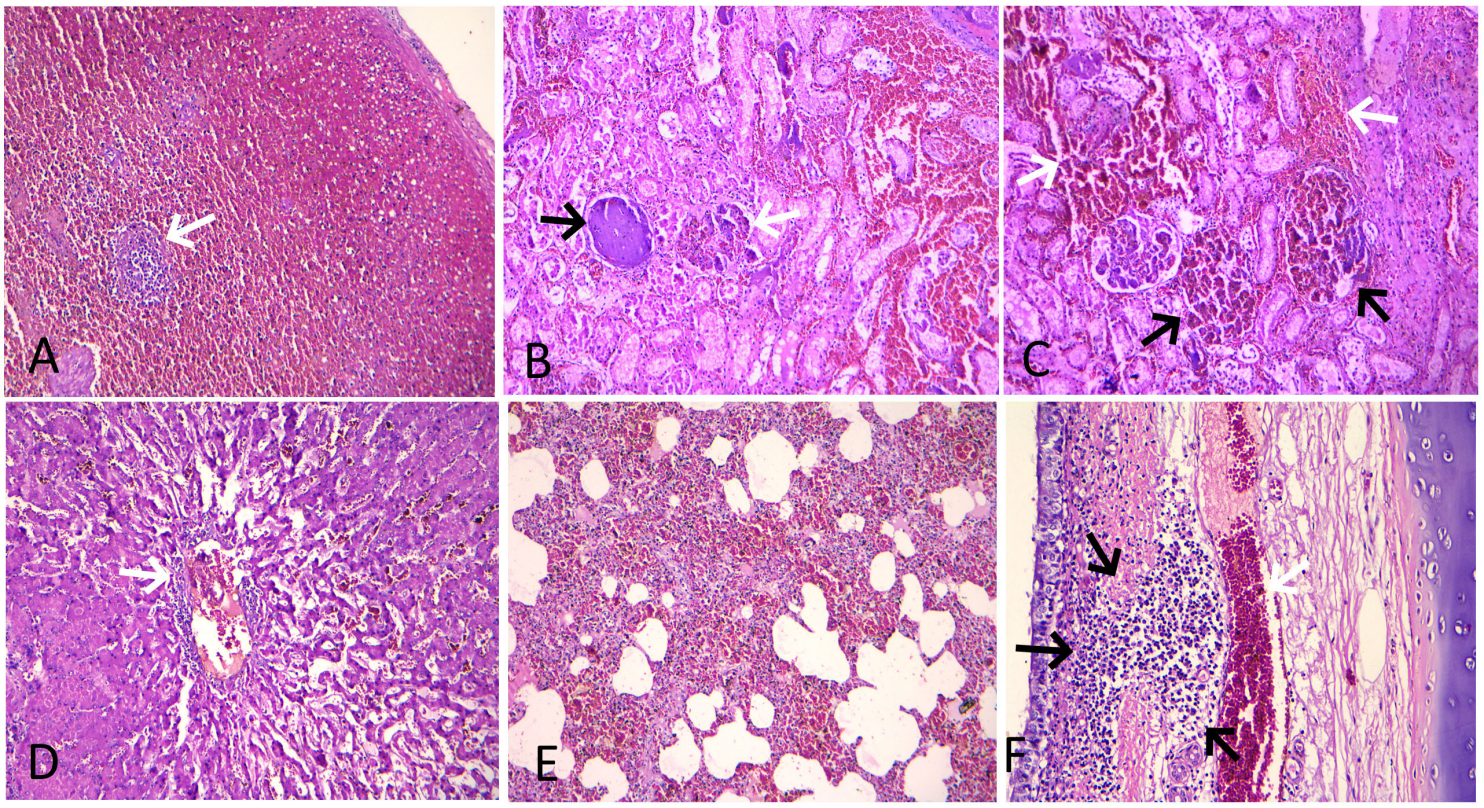
Histopathological lesions in ASF-infected pigs. **(A)** Spleen showing severe hyperemia with abundant red blood cells in the red pulp and severe lymphoid depletion with atrophy of the lymphoid follicle (arrow) in the white pulp (H&E stain, × 100). **(B)** Kidney showing hyperemia of the capillary tufts, necrosis, and marked reduction in Bowman’s space in a glomerulus (white arrow), along with a globally sclerotic (obsolescent) glomerulus (black arrow). An area of severe hyperemia and hemorrhage within the intertubular spaces is also visible on the right (H&E stain, × 200). **(C)** Kidney showing severe hyperemia and hemorrhage in the interstitial (white arrow) and glomerular capillaries (black arrow) (H&E stain, × 200). **(D)** Liver showing dilatation and congestion of central veins and sinusoidal capillaries, along with necrotic and degenerative changes in hepatocytes. Also note perivascular lymphocyte infiltration and the presence of cellular debris and erythrocytes within the central vein (arrow) (H&E stain, × 200). **(E)** Lung showing thickened alveolar wall due to mononuclear cellular infiltration and hemorrhages in the septa (H&E stain, × 100). **(F)** Trachea showing moderate infiltration of lymphocytes in the lamina propria of tracheal mucosa (black arrow) and severely engorged blood capillaries (white arrow) (H&E stain, × 200).

Microscopic lesions in the spleen were characterized by severe hyperemia with multifocal hemorrhages in the red pulp area, whereas severe lymphoid depletion with atrophy of lymphoid follicles was observed in the white pulp (**Figure 4A**). In addition, necrosis of lymphoid cells around germinal centers and fibrinoid necrosis of small arterioles in the spleen were observed in four cases. The kidneys showed consistent histopathological lesions, including hyperemic blood vessels and hemorrhages in the intertubular spaces within the renal cortex (**Figure 4B**). The majority of the glomeruli exhibited necrosis, distortion, hyperemia of the capillary tufts, hemorrhages, marked reduction in Bowman’s space, and focal and segmental glomerulosclerosis (**Figures 4B, C**). The renal tubules showed diffuse necrosis and degeneration, with many filled with proteinaceous fluid. The mesenteric lymph nodes showed moderate lymphoid depletion in the lymphoid follicles, along with focal hemorrhages in the cortical region in three cases. The liver showed dilatation and congestion of central veins and hyperemia of sinusoidal capillaries, along with necrotic and degenerative changes in hepatocytes. In addition, lymphocyte infiltration around the central veins, with the presence of cellular debris and erythrocytes, was observed (**Figure 4D**). Infiltration of lymphocytes and fibrous connective tissue around hepatic lobules was also observed in four cases. Histopathology of the lung showed interstitial pneumonia characterized by thickened alveolar and bronchial walls due to mononuclear cellular infiltration—mainly lymphocytes and macrophages—and hemorrhages in the septa (**Figure 4E**). In addition, edema and emphysema were observed in the lungs of infected pigs. The trachea showed moderate infiltration of lymphocytes in the lamina propria of the tracheal mucosa and severely engorged blood capillaries (**Figure 4F**). Histopathology of the heart revealed moderate to severe hyperemia of capillaries in the myocardium in all cases, whereas focal hemorrhages were observed in three cases. The small intestine exhibited severe congestion of blood vessels in the lamina propria of the mucosa, focal hemorrhages, and increased mucosal width.

### 3.3 Molecular detection and phylogenetic analysis

**Figure 5:**
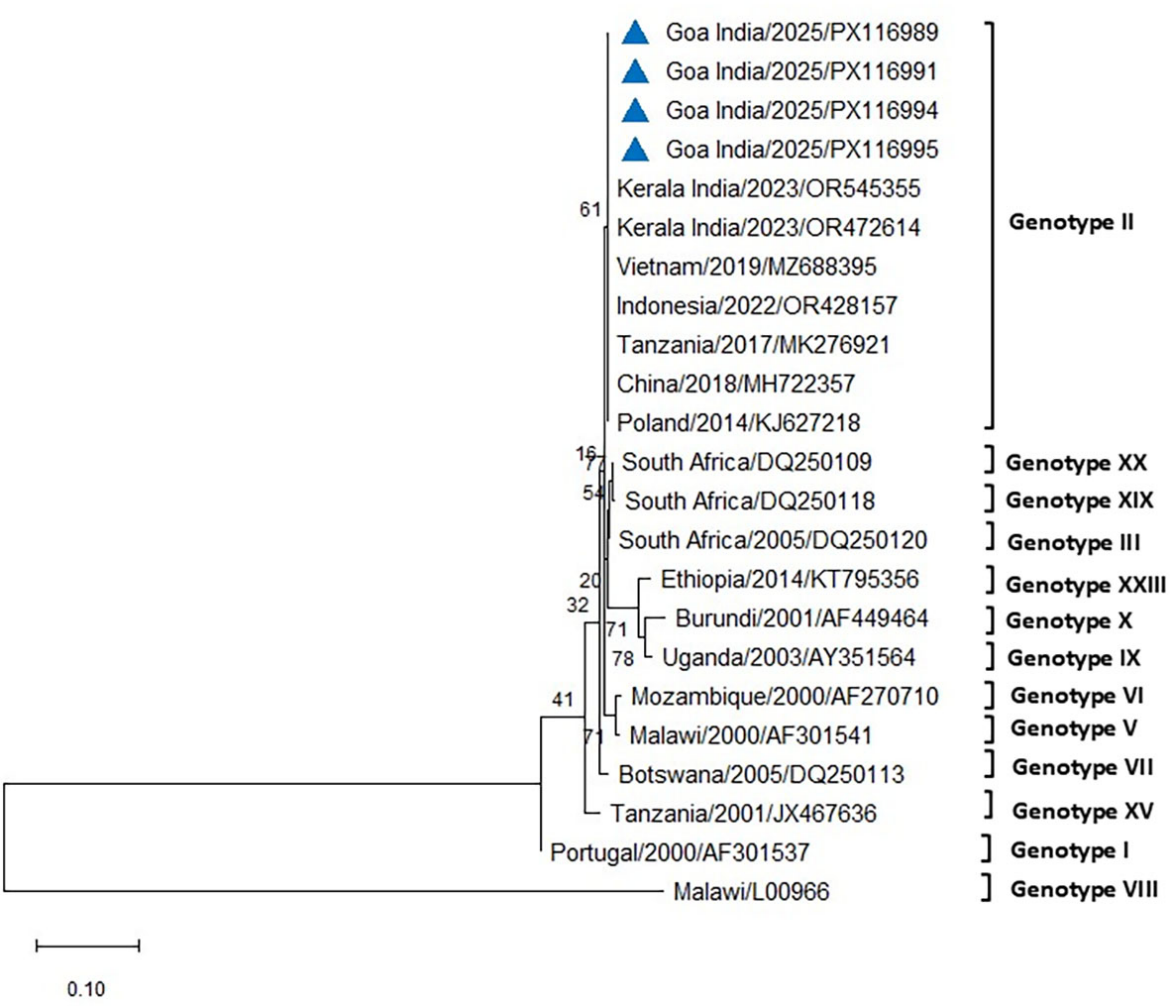
Phylogenetic analysis based on partial p72 gene sequences of ASFV from two farms in Goa, India (triangle mark), and other ASFV reference sequences obtained from NCBI Genbank. The country name is followed by the year of outbreak and GenBank accession number. Multiple sequence alignments were performed using the Clustal W tool in MEGA12, and a phylogenetic tree was constructed using the Neighbor-Joining method with the Tamura–Nei model and 1,000 bootstrap replicates.

**Figure 6:**
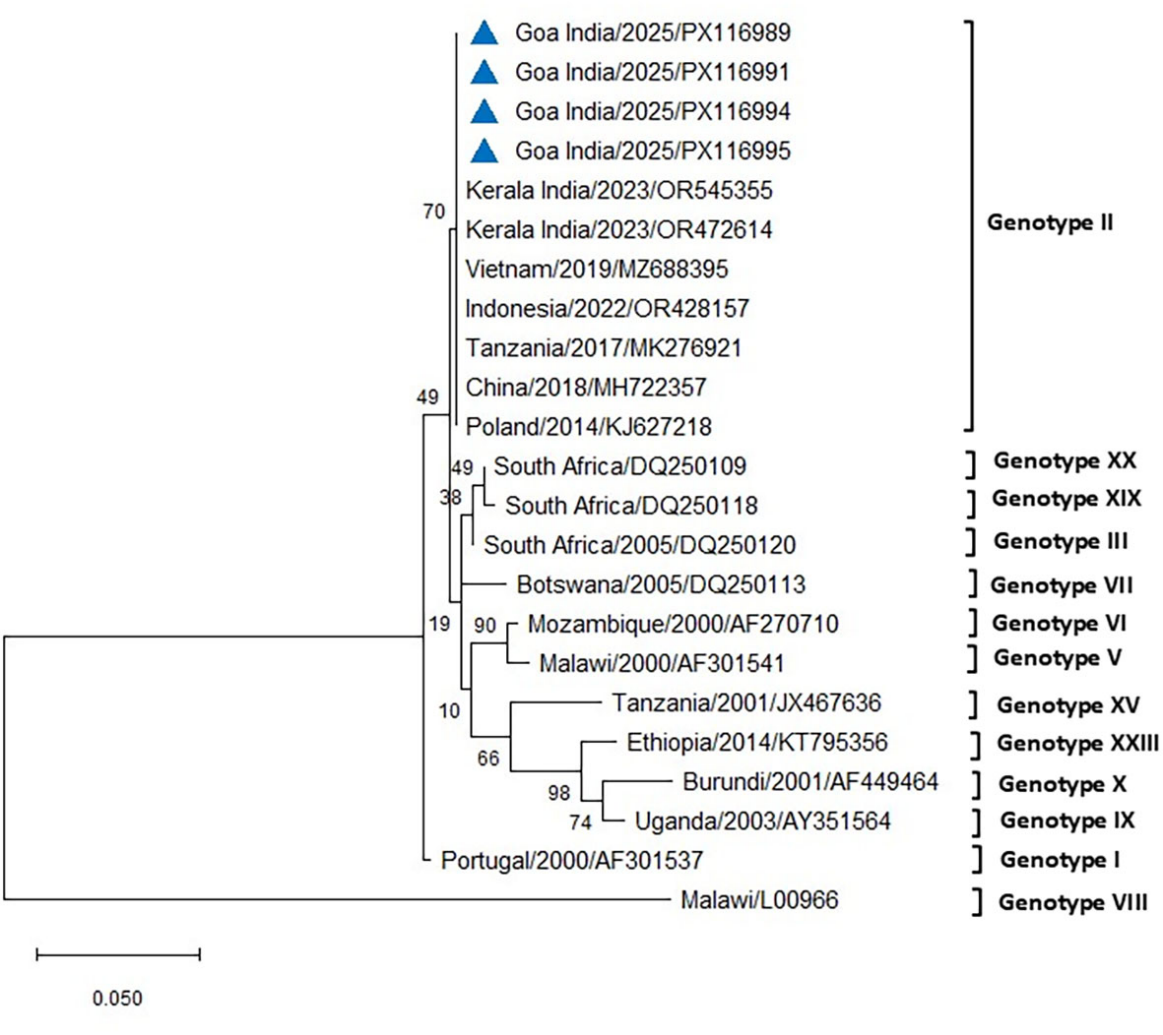
Phylogenetic analysis based on partial p72 gene sequences of ASFV from two farms in Goa, India (triangle mark), and other ASFV reference sequences obtained from NCBI Genbank. The country name is followed by the year of outbreak and GenBank accession number. Multiple sequence alignments were performed using the Clustal W tool in MEGA12, and a phylogenetic tree was constructed using the maximum likelihood method with the Tamura–Nei model and 1,000 bootstrap replicates.

The initial diagnostic PCR targeting the 257-bp *B646L* gene confirmed the presence of ASFV DNA in all tissues and blood samples of the tested infected pigs. The eight representative sequences of the *B646L* gene were also deposited in NCBI GenBank, with accession numbers assigned (PV837539–PV837546). BLAST analysis of this *B646L* gene in NCBI revealed 100% nucleotide identity with the available ASFV sequences in GenBank. The PCR for the p72 gene successfully amplified ASFV from all the blood and tissue samples from the infected pigs. The 478-bp region of the *B646L* gene encoding the major viral capsid protein p72 was amplified from nine positive cases, subsequently sequenced, analyzed, and submitted to GenBank (accession numbers PX116989–PX116997). These sequences were compared with seven previously defined reference sequences of genotype II, along with other major ASFV genotypes I, III, V, VI, VII, VIII, IX, X, XV, XIX, and XX, to classify the viruses associated with the outbreaks in the two farms in Goa. Comparative analysis of the ASFV p72 nucleotide sequences from Goa with reference genotype II sequences from Tanzania, Indonesia, Poland, China, Vietnam, and a recent outbreak sequence from Kerala, India, revealed 100% identity using both Neighbor-Joining and maximum likelihood methods (**Figures 5**, **6**).

**Figure 7:**
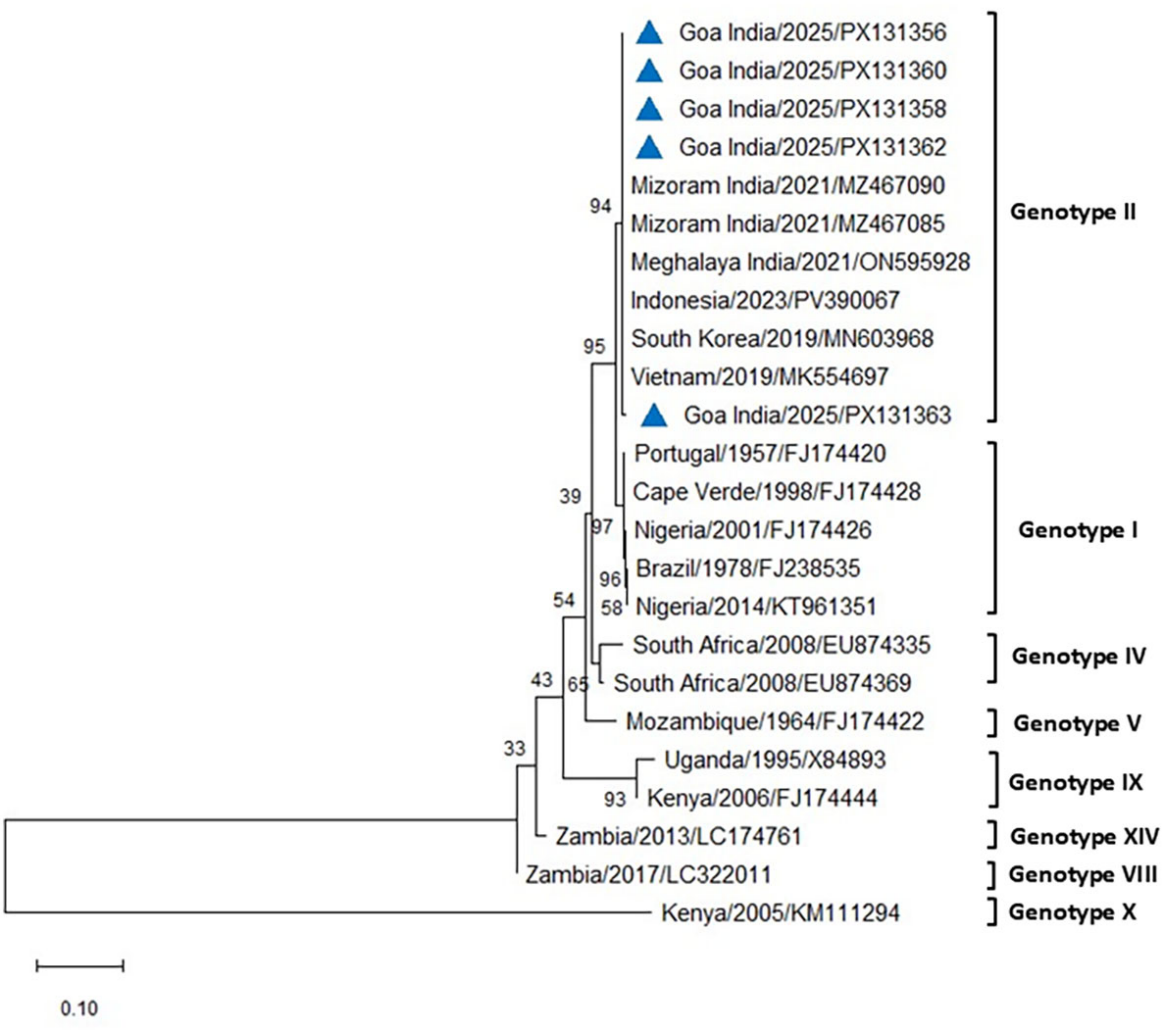
Phylogenetic analysis based on the *E183L* gene encoding p54. Sequences of ASFV from two farms in Goa, India (triangle mark), were compared with other ASFV reference sequences obtained from NCBI Genbank. The country name is followed by the year of outbreak and GenBank accession number. Multiple sequence alignments were performed using the Clustal W tool in MEGA12, and a phylogenetic tree was constructed using the Neighbor-Joining method with the Tamura–Nei model and 1,000 bootstrap replicates.

**Figure 8:**
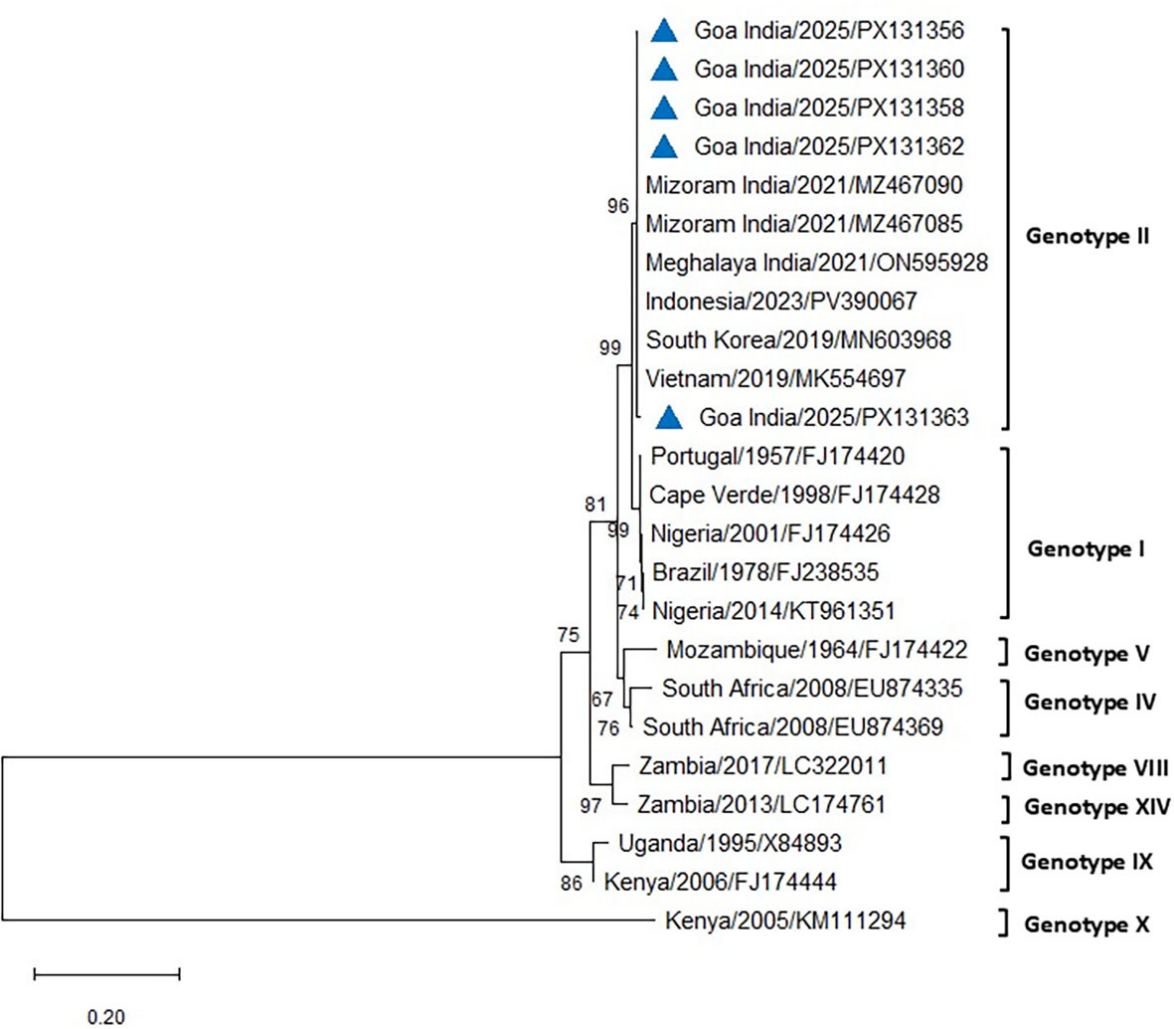
Phylogenetic analysis based on the *E183L* gene encoding p54. Sequences of ASFV from two farms in Goa, India (triangle mark), were compared with other ASFV reference sequences obtained from NCBI Genbank. The country name is followed by the year of outbreak and GenBank accession number. Multiple sequence alignments were performed using the Clustal W tool in MEGA12, and a phylogenetic tree was constructed using the maximum likelihood method with the Tamura–Nei model and 1,000 bootstrap replicates.

The PCR for the p54 gene successfully amplified ASFV from all the blood and tissue samples from the infected pigs of both farms. The amplified 683-bp region of the *E183L* gene encoding the p54 protein from eight positive cases was sequenced, analyzed, and submitted to GenBank (accession numbers PX131356–PX131363). Comparative sequence analysis of p54 PCR products obtained from five different cases across the two farms in Goa, India, produced results similar to those obtained using p72 and revealed 100% similarity with reference sequences of genotype II from Indonesia, South Korea, Vietnam, and the Mizoram and Meghalaya regions of India, using both Neighbor-Joining and maximum likelihood methods (**Figures 7**, **8**). All DNA samples tested by PCR against PCV2, *E. coli*, *Pasteurella multocida*, *Streptococcus* sp., and CSFV tested negative.

## 4 Discussion

Since the first report of ASF outbreaks in the north-eastern states of India in January 2020, repeated occurrences have been documented, raising concerns that the disease may now be endemic in this region (Rajkhowa et al., 2024; Times of India, 2025). Despite the implementation of government-mandated regulations and control measures, sporadic outbreaks continue to be reported from regions of the country previously considered unaffected. Notably, ASF was confirmed in a pig farm in Ghaziabad, Uttar Pradesh, in February 2023, and in four pig farms in Kannur district, Kerala, in March 2023, representing the first documented occurrences in the northern and southern regions of India, respectively (Hiremath et al., 2024; Verma et al., 2024). It is plausible that, following these outbreaks, sporadic cases in smallholder and backyard pig farms with minimal biosecurity facilitated further local spread. Such outbreaks may remain unnoticed or unreported due to low pig population sizes and limited surveillance. The present investigation documents the first confirmed occurrence of ASF in two pig farms in Goa, representing the entry of the virus into the west coast region of India. The outbreak was characterized by high mortality, rapid transmission, and severe acute pathological lesions, underscoring the urgent need for stringent preventive and control strategies to curtail disease spread and mitigate economic losses among pig farmers.

The clinical manifestations observed in affected pigs from both farms were consistent with previously reported ASF symptoms (Salguero, 2020; Rajkhowa et al., 2022; Hiremath et al., 2024). Notably, subcutaneous hemorrhages appeared more prominent and widespread in the white-coated LWY breed compared to black or dark-colored local and crossbred pigs, suggesting potential breed-specific differences in susceptibility and lesion severity that warrant further investigation. Nevertheless, in the present study, the overall mortality pattern and severity of visceral lesions were comparable between the two breeds. Respiratory distress accompanied by nasal discharge, as recorded in the present outbreak, aligns with descriptions of highly pathogenic ASFV infections in the acute form (Carrasco et al., 2002; Salguero, 2020). In contrast, clinical signs such as vomiting and diarrhea, reported in some earlier studies (Sánchez-Vizcaíno et al., 2015; Verma et al., 2024), were not observed in these cases. Consistent with observations from naïve farms in previous reports, the acute clinical form predominated in the present study, most likely attributable to infection with highly or moderately virulent ASFV isolates (Salguero, 2020). In the acute form of ASF, mortality in affected farms may reach nearly 100% within a week of clinical onset, reflecting the highly virulent nature of the infection (Salguero, 2020). Our study also recorded approximately 95% to 96% mortality, indicating involvement of highly or moderately virulent ASFV. However, during the first report of ASF in the north-eastern region of India in 2020, considerably lower morbidity (38.45%) and mortality (33.89%) were recorded, indicating that previous Indian ASF isolates were less virulent and subacute (WOAH, 2020).

The gross lesions, such as splenomegaly and hepatomegaly with severe hyperemic and hemorrhagic lesions in multiple internal organs—including kidneys, lungs, liver, heart, and intestines—were more consistent with lesions of acute ASF described in many earlier studies (Salguero et al., 2002; Salguero, 2020; Pegu et al., 2023). Similarly, the histopathological lesions, including multifocal to diffuse hemorrhages in multiple organs, lymphoid depletion and necrosis in spleen and lymph nodes, diffuse hemorrhages with mononuclear cell infiltration in the lung and liver, and acute proliferative glomerulonephritis, were consistent with the acute form of ASF (Hervás et al., 1996; Salguero et al., 2002; Izzati et al., 2020; Salguero, 2020; Pegu et al., 2023; Hiremath et al., 2024). Lesions of acute proliferative glomerulonephritis and tubular necrosis are associated with viral replication within mesangial cells and collecting ducts (Hervás et al., 1996). Similarly, widespread hemorrhages have been linked with endothelial dysfunction, which is further exacerbated by viral replication in endothelial cells as well as in the smooth-muscle cells of arterioles and venules (Hervás et al., 1996). This indicates that vascular injury plays a key role in disease progression, as damage to both endothelial integrity and vascular tone can act together to worsen tissue damage and increase disease severity. Overall, the clinical signs, mortality pattern, and gross and histopathological lesions observed in the infected pigs of this study were characteristic of an acute form of infection.

Effective mitigation and management of outbreaks require the timely and accurate identification of infection sources and transmission routes, followed by the rapid implementation of control measures. ASFV is regarded as highly stable and can spread efficiently through infected pigs, contaminated pig products during trade, or via blood-feeding by infected Ornithodoros ticks (Lv et al., 2022). Although the role of Ornithodoros soft ticks in ASFV transmission—through biological, mechanical, or passive routes—has been reported in Africa, Europe, and the USA (Burrage, 2013; Forth et al., 2020; Lv et al., 2022), the present study did not detect any evidence of tick infestation in the affected farms. However, given that such transmission has not been documented in India or elsewhere in Asia to date (Patil et al., 2020), the possibility of their involvement in local ASFV spread cannot be ruled out and requires cautious, detailed investigation. In farm A, swill feeding with poultry slaughter waste was practiced, which may have served as a potential source of ASFV introduction. The swill collection and distribution vehicle reportedly visited multiple pig farms, although no records were available, making it plausible that the virus could have been introduced from an infected source via swill feeding. Swill feeding is recognized as a major risk factor for ASF propagation, with reports estimating that outbreaks linked to swill can result in a five-fold higher epidemic peak and up to seven-fold greater pig mortalities compared to farms without such practices (Li et al., 2024). By contrast, swill feeding was not practiced in farm B, yet the observed mortality was comparable to that in farm A. The nearest pig unit to farm A was an organized intensive farm located approximately 3 km away, where swill feeding was not practiced and strict biosecurity measures were implemented following confirmation of ASF in farm A. To date, no ASF outbreak has been reported from this farm, underscoring the critical role of biosecurity and the avoidance of swill feeding in safeguarding naïve pig populations.

The phylogenetic analysis of ASFV isolates from the present study indicated clustering within genotype II, which remains the only genotype reported from India to date (Rajukumar et al., 2021; Rajkhowa et al., 2022; Buragohain et al., 2023; Hiremath et al., 2024). Gene-based analyses using p72 and p54 markers are widely considered useful for monitoring genetic variation and exploring potential transmission dynamics of ASFV in new regions (O’Dwyer et al., 2025). Whole-genome sequencing, however, provides higher resolution by detecting both neutral and functional mutations across the viral genome, thereby contributing to a more comprehensive understanding of ASFV evolution and epidemiology (O’Dwyer et al., 2025). The isolates examined in this study showed close phylogenetic relatedness to genotype II strains reported previously from India and other Asian countries. While this finding confirms the continued circulation of genotype II in the region, the precise origins and transmission routes of the virus in the present outbreak remain unresolved. The 100% nucleotide sequence similarity in the p72 region between the Goa isolate and a recent genotype II strain from an outbreak in Kerala raises the possibility of an epidemiological linkage. However, as the precise source and route of transmission could not be established, this observation should be interpreted with caution, with circumstantial evidence only tentatively pointing to the potential role of pig movement or pork product trade between Kerala and Goa. Further investigations, including whole-genome sequencing and epidemiological studies, may provide additional insights into the pathways of introduction and spread.

Although co-infections of ASFV with two or more pathogens have been frequently documented in pig herds elsewhere (Zhao et al., 2021), including recent reports of ASFV co-infection with highly pathogenic PRRSV in India (Rajkhowa et al., 2024), no such co-infections were identified in the present investigation. Nevertheless, the potential for future ASF outbreaks to occur in conjunction with other virulent pathogens remains a serious concern, as such co-infections could exacerbate mortality rates and amplify economic losses.

## 5 Conclusion

In conclusion, the first detection of ASF in the west coast region of India underscores the need to strengthen biosecurity measures, improve farmer awareness, and implement stringent surveillance to reduce the risk of establishment and further spread of this disease to other pig-rearing regions of the country.

## Data Availability

The datasets presented in this study can be found in online repositories. The names of the repository/repositories and accession number(s) can be found in the article/Supplementary Material.
